# Leaf Spectral Reflectance Shows *Thalassia testudinum* Seedlings More Sensitive to Hypersalinity than Hyposalinity

**DOI:** 10.3389/fpls.2017.01127

**Published:** 2017-06-28

**Authors:** Michael J. Durako, Jacqueline F. Howarth

**Affiliations:** Department of Biology and Marine Biology, Center for Marine Science, The University of North Carolina Wilmington, WilmingtonNC, United States

**Keywords:** *Thalassia testudinum*, seedlings, spectral reflectance, salinity, stress

## Abstract

*Thalassia testudinum* (turtle grass) is the dominant and climax-successional seagrass species in the subtropical/tropical Atlantic and Caribbean region. Two die-offs of *T. testudinum* in Florida Bay, United States have raised concerns regarding the resilience of this species to environmental disturbances. Seedlings are important in recovery of *T. testudinum*, following disturbance events. Leaf spectral reflectance [*R*(λ)] was measured in *T. testudinum* seedlings exposed for 2 weeks to three salinities (20, 35, and 50) and two light levels (full sun and 50–70% light reduction) in experimental mesocosms. Multivariate analyses indicated that hypersalinity had a greater effect on spectral reflectance than hyposalinity or light reduction. There was an increase in variability and flattening of reflectance spectra at the highest salinity. All three salinity treatments had distinct reflectance spectra across green wavelengths (530–580 nm), with additional discrimination between 20 versus 50 and 35 versus 50 treatments across red wavelengths (630–690 nm). Red:Green reflectance ratios were highest and photochemical reflective index values were lowest for the salinity 50 treatment, but were not significantly different between the salinity 20 and 35 treatments. The changes in the *R*(λ) spectra for the salinity 50 seedlings were consistent with previously observed reductions in leaf pigments and maximum photochemical efficiency of photosystem II. These observations indicate that leaf spectral reflectance is a sensitive indicator of plant stress in *T. testudinum* seedlings and that seedlings are more sensitive to short-term exposures to hypersalinity than hyposalinity.

## Introduction

*Thalassia testudinum* Banks ex König (turtle grass) is the dominant seagrass species in the subtropical/tropical Atlantic and Caribbean region (den Hartog, 1970; Green and Short, 2003). This species has high light requirements and is considered to be the climax species in marine seagrass systems (Zieman, 1982). *T. testudinum* is also the dominant seagrass and dominant physical structure in Florida Bay, a shallow (<2 m, Schomer and Drew, 1982) subtropical estuary that exhibits wide variations in salinity and water clarity (Zieman, 1982; Hall et al., 1999; Durako et al., 2002). Two massive die-offs of *T. testudinum* have occurred in Florida Bay, United States, the first in 1987–1990 (Robblee et al., 1991) and more recently in late summer 2015 (Hall et al., 2016). Several years after the 1987–1990 die-off, Florida Bay began exhibiting widespread turbid conditions due to phytoplankton blooms and resuspended sediments (Phlips and Badylak, 1996; Stumpf et al., 1999). This increase in turbidity was associated with additional light-stress-induced losses of *T. testudinum* in western and central Florida Bay (Durako et al., 2002). The two die-off events were nearly equivalent in terms of size, locations, seasonality, speed and appearance (Hall et al., 2016) suggesting common underlying causative factors. Hypersalinity, elevated water temperatures and water column stratification leading to bottom-water anoxia, hypoxic stress and sulfide toxicity are thought to have lead to increased stress and subsequent mortality of very dense *T. testudinum* (Hall et al., 2016).

In response to the 1987–1990 die-off, the Comprehensive Everglades Restoration Program (CERP) was initiated to increase freshwater inflow to Florida Bay back to historical patterns (Herbert et al., 2011). Decreased salinity is a major management goal of CERP, which may affect seagrass species recovery patterns. Fourqurean et al. (2003) predicted that the more estuarine seagrasses, such as *Halodule wrightii* and *Ruppia maritima*, would increase in abundance at the expense of the marine *T. testudinum*. Because seagrasses are the dominant benthic community in Florida Bay, changes in their species distributions and recruitment patterns are being used as a central performance measure to assess CERP success.

Seedlings are an important source of recruitment in recovery of *T. testudinum*, following disturbance events (Whitfield et al., 2004). Because *T. testudinum* exhibits continuous embryo development, germinating as embryos are released from parent plants, there is no seed bank (Kuo and den Hartog, 2006). Thus, seedlings physiological health, survival and growth are dependent on the environmental conditions existing during the release of the annual cohorts. Because *T. testudinum* exhibits monopodial clonal growth (Tomlinson and Vargo, 1966), the use of field-collected short shoots rather than seedlings in experimental studies could result in pseudoreplication. This makes seedlings ideal independent experimental units on which to assess the effects of light and salinity. In a previous examination of diurnal variation in chlorophyll fluorescence of *T. testudinum* seedlings in response to variations in salinity and light, we observed significant reductions in photochemical efficiency of photosystem II (*F*_v_*/F*_m_) under hypersaline (salinity 50), but not hyposaline (salinity 20) conditions, suggesting a greater sensitivity to high-salinity stress (Howarth and Durako, 2013a). In contrast, significant increases in *F*_v_*/F*_m_ in shaded seedlings indicated low-light acclimation. Seedlings in the salinity 50 treatment also had significantly reduced chlorophyll and carotenoid contents and increased non-photochemical quenching (NPQ) compared to the salinity 20 and 35 treatment seedlings, while shading resulted in increases in chlorophyll *b* resulting in significant reductions in chlorophyll *a:b* ratios (Howarth and Durako, 2013b).

The measurement of spectral reflectance [*R*(λ)] is a non-destructive optical technique that has long been used as an indicator of plant stress (Carter and Knapp, 2001). Leaf *R*(λ) is an apparent bio-optical property that varies largely in response to changes in leaf pigments (Sims and Gamon, 2002). There are several spectral-reflectance indices based on reflectance at specific regions of the spectrum, which may be used to predict relative leaf pigment contents (Sims and Gamon, 2002). Changes in pigments may reflect plant stress and affect the ability of seagrasses to tolerate variations in light through the regulation of energy transduction (Dennison and Alberte, 1986; Beer et al., 2000; Ralph et al., 2002; Belshe et al., 2007). For example, carotenoids may contribute to photosynthesis as accessory pigments or have photoprotective functions (Ralph et al., 2002). Under conditions of excess irradiance and hypersalinity, carotenoids that comprise the xanthophyll cycle will dissipate excess energy, which avoids damaging photosystems (Demmig-Adams et al., 1996; Howarth and Durako, 2013b). Anthocyanins may also protect leaves from excess visible or UV light or they may serve as antioxidants (Gould et al., 2002). Because pigments serve either photosynthetic or photoprotective functions, changes in *R*(λ) may correspond with changes in relative photosynthetic efficiency and capacity under various environmental conditions (Bargain et al., 2013). Pigment reductions and increased spectral reflectance are typical responses to plant stress (Carter and Knapp, 2001). However, turbid low-light conditions can result in increases in leaf chlorophyll content and a reduction in the chlorophyll *a* to *b* ratio (Dennison and Alberte, 1985; Longstaff and Dennison, 1999; Cummings and Zimmerman, 2003). These changes in chlorophyll contents increase the amount of light absorbed for photosynthesis and thus, reduce reflectance, especially in the blue (400–500 nm) and red (660–680 nm) regions of the spectrum (Sims and Gamon, 2002). In addition to being non-destructive, measurement of *R*(λ) is rapid and can be applied across multiple spatial scales (Gamon and Qui, 1999), which is why it has been widely used as an indicator of plant health for terrestrial plants (Carter and Knapp, 2001; Sims and Gamon, 2002). In contrast, this methodology has had very limited application in quantifying stress responses in seagrasses (Thorhaug et al., 2006). Here, we report on variations in leaf spectral reflectance of the *T. testudinum* seedlings in response to 2 weeks exposure to three salinity treatments (20, 35, and 50) and two light treatments (full sun and 50–70% reduction from ambient light). Based on our previous observations on photochemical efficiency and pigment contents (Howarth and Durako, 2013a,b), we hypothesized that *R*(λ) would increase in response to hypersalinity, but decrease under shaded conditions.

## Materials and Methods

### Sample Collections

Seedlings of *T. testudinum* collected from Key Biscayne, Florida (25.716^o^ N 80.149^o^ W) were shipped overnight to the University of North Carolina Wilmington, Center for Marine Science (UNCW/CMS), Wilmington, North Carolina. After arrival to CMS, the seedlings were immediately planted in six-celled plastic nursery pots (each cell 5 cm × 5 cm × 7 cm) containing aragonite shell hash and they were held in a holding vault (55 cm × 110 cm × 30 cm) with flow-through seawater (salinity 29–35). The Practical Salinity Scale (PSS) was used to determine salinity. Units are not assigned to salinity values because it is a ratio and has no units as defined by UNESCO (1985). Seedlings were allowed to grow for 4 weeks in the vault prior to their placement in the experimental aquaria.

### Mesocosm Experiment

**FIGURE 1:**
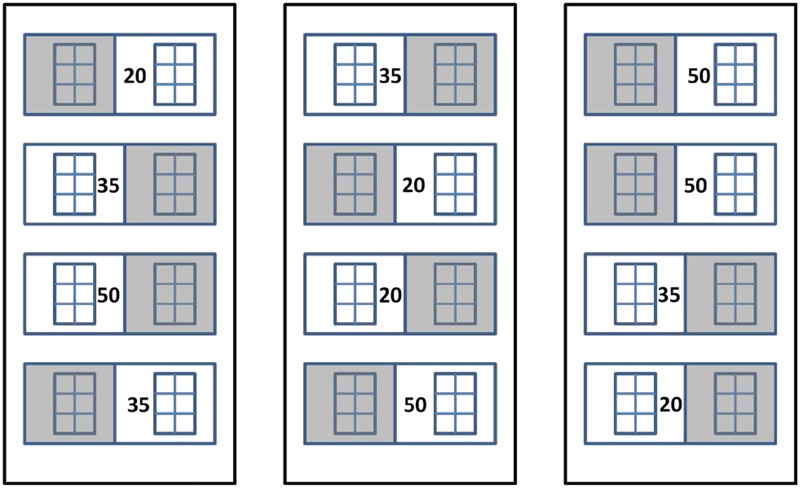
Experimental design for *Thalassia testudinum* seedling mesocosm experiment. Numbers represent salinity treatment values and shaded squares represent 50–70% shade cloth (*n* = 4).

At the start of the experiment, two six-celled pots were placed in each of four replicate 38-l treatment aquaria (*n* = 4). Aquaria were placed outside within seawater-supplied fiberglass vaults (55 × 110 × 30 cm) located on the south side of CMS (**Figure 1**). The vaults acted as water baths to minimize daily water temperature fluctuations, and the aquaria were randomly arranged within the vaults (four aquaria per vault) to account for spatial differences. The experimental design consisted of two light treatments (full sun = Sun and 50–70% shade = Shade) and three salinity treatments (20, 35, and 50). Initially, all seedlings were acclimated to the control salinity (35) under full sun for 7 days. Following acclimation, neutral-density screens (2 layers of 30% nursery shade cloth) were placed over the shaded treatment seedlings and either de-ionized (DI) water or Instant Ocean (IO) salts were added to the appropriate aquaria to decrease or increase the salinity by 2 per day until target salinities were reached 8 days later. The gradual change in salinity allowed seedlings time to acclimate to the changes in salinity (Kahn and Durako, 2006). Salinities were checked daily with a YSI Model 80 conductivity meter (Yellow Springs, OH, United States) for the duration of the experiment and adjusted with either DI of IO salts. Photosynthetically active radiation (PAR) was measured at the plant level using underwater scalar quantum sensors (LiCor LI-193S) attached to a LiCor LI-1400 datalogger (Lincoln, NE, United States). PAR was measured every 15 min. During the experiment, leaf blades were gently wiped to remove epiphytes and detritus six times per day (at 0600, 0900, 1200, 1500, 1800, and 2100 h). Leaf samples for pigment analyses (reported in Howarth and Durako, 2013b) and leaf spectral reflectance measurements were obtained after the seedlings had been at target salinities for 14 days.

### Spectral Reflectance

Spectral reflectance [*R*(λ)] is the fraction (%) of downwelling plane irradiance that reflects upward as a function of wavelength (λ) [*R*(λ) = *E*_u_(λ)*/E*_d_(λ)]. Reflectance measurements employed an Ocean Optics USB2000 fiber optic spectrometer coupled to a 400 μm diameter UV-VIS reflection probe (Ocean Optics R400-7-UV-VIS). To measure upwelling irradiance [*E*_u_(λ)], the probe was positioned 0.5 cm above the surface of each rank 2 leaf at a 45^o^ zenith angle using a clear lexan leaf clip. Immediately following the upwelling irradiance measurement, downwelling irradiance [*E*_d_(λ)] was measured using a Spectralon diffuse reflectance target (Ocean Optics WS-1) placed adjacent to, and in the same plane as, the leaf. It was assumed that the Spectralon target is Lambertian (i.e., reflects light equally in all directions). Ten spectra per sample were averaged and then smoothed with a 5 nm boxcar filter window to ensure an optimal signal-to-noise ratio and preserve the spectral features (Fyfe, 2003).

### Statistical Analyses

Because near infrared wavelengths are absorbed by water and cannot by assessed by remote sensing through the water column, reflectance spectra data from only the 400 to 700 nm range were processed (Fyfe, 2003). To facilitate among-treatment comparisons of reflectance spectra without the effects of spectral magnitudes, spectra were normalized by dividing each wavelength specific reflectance value by the overall mean reflectance from 400 to 700 nm for that leaf (Hochberg et al., 2004). The magnitude of among-treatment variation in normalized spectral reflectance was compared using multivariate analyses in PRIMER version 6 (Clarke and Warwick, 2001). A similarity matrix was generated for the normalized reflectance spectra with Bray-Curtis and non-metric multidimensional scaling (nMDS) two-dimensional ordinations were used to visualize treatment relationships. The use of nMDS allowed the entire normalized reflectance spectra for each replicate seedling to be compared within and among each of the experimental treatments. To test hypotheses that the normalized reflectance spectra differed among salinities and between light treatments, a non-parametric two-way analysis of similarity (ANOSIM) was performed. ANOSIM uses Bray-Curtis similarities to compare ranks of between-group to within-group similarities to test the null hypothesis that the groups do not differ. Similarity percentage (SIMPER) procedures were performed to identify which wavelengths contributed the most to the differences among salinities or between light treatments. Wavelengths with high percentage contributions to dissimilarities can be considered good for discriminating among treatments (Fyfe, 2003). Based on the SIMPER results, two reflectance indices were calculated: (1) the red:green ratio (=*R*_600-699_*/R*_500-599_), which provides an effective estimate of the ratio of anthocyanins to chlorophyll (Sims and Gamon, 2002), and (2) the photochemical reflectance index [PRI = (*R*_531_-*R*_570_)/(*R*_531_+*R*_570_)], which is an indicator of epoxidation of xanthophylls cycle pigments and photochemical efficiency (Gamon et al., 1997). Among-treatment variation in the red:green ratio was assessed with Kruskal Wallis 1-Way ANOVA on ranks followed by Student-Neuman-Kuels (SNK) multiple pairwise comparisons. Among-treatment in the PRI was assessed by a 1-Way ANOVA followed by SNK multiple pairwise comparisons. Significance was determined at *p* < 0.05.

## Results

**FIGURE 2:**
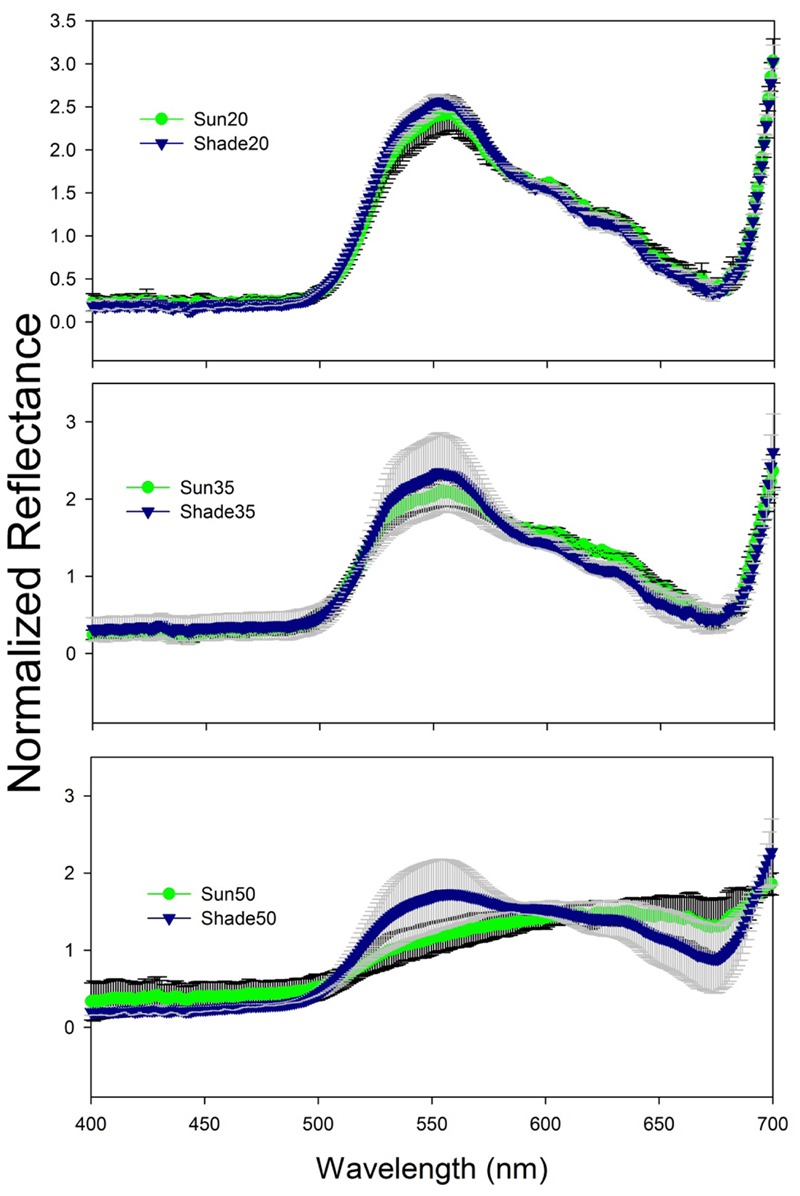
Spectral reflectance (mean ± s.d.) for *T. testudinum* seedlings leaves after 14 days exposure to salinity 20 (top), 35 (middle), and 50 (bottom) treatments in full sun (Sun, green line with black error bars) or under 50–70% light reduction (Shade, blue line with gray error bars; *n* = 4).

**FIGURE 3:**
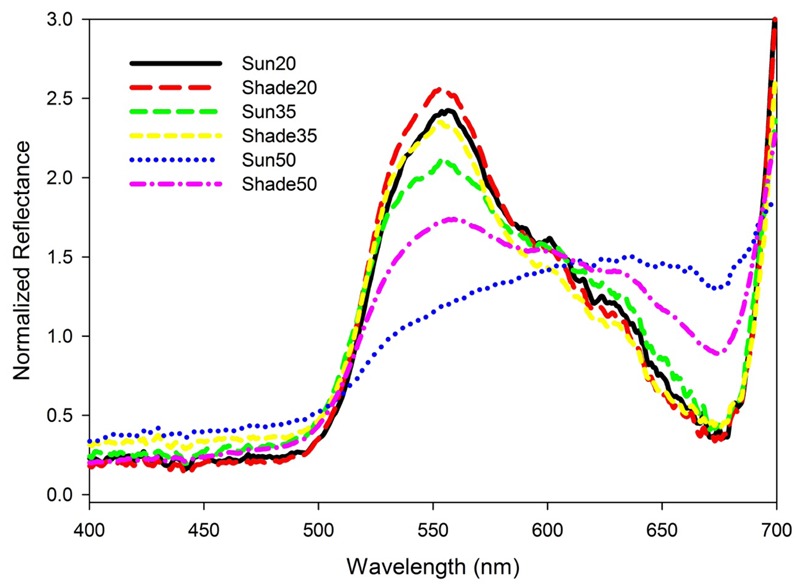
Mean spectral reflectance for *T. testudinum* seedlings leaves after 14 days exposure to salinity 20, 35, and 50 treatments in full sun (Sun) or under 50–70% light reduction (Shade; *n* = 4).

Normalized reflectance spectra for the 20 and 35 salinity treatment leaves were typical of green vascular plant leaves containing chlorophylls *a* and *b* (**Figure 2**). Reflectance was relatively low and constant from 400 to 500 nm then increased toward a peak around 550 nm followed by a decrease to another low around 675 nm and then rapidly increasing to 700 nm. There was an increase in variability among spectra as salinity increased (**Figure 2**) and a flattening of spectral reflectance at the highest salinity (**Figure 3**). Shade-treatment seedlings generally exhibited higher reflectance between 525 to 575 nm and lower reflectance around 675 nm compared to the unshaded (Sun) treatment.

**FIGURE 4:**
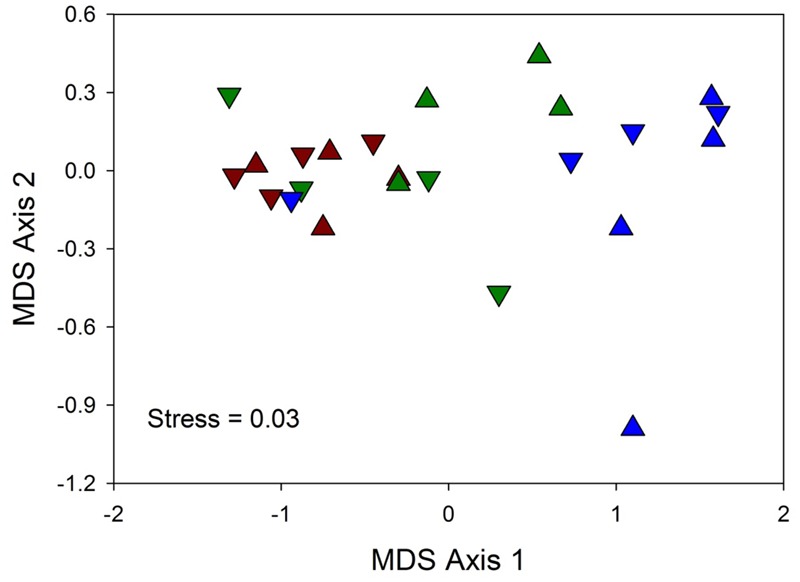
Two-dimensional MDS ordination of reflectance spectra similarity matrices among three salinity treatments (20 = red, 35 = green, and 50 = blue) and two light treatments (▲ = Sun, and ▼ = Shade) for four replicate (R1-R4) *T. testudinum* seedlings leaves after 14 days exposure (*n* = 4).

Multivariate (nMDS) ordination indicated that hypersalinity had a greater effect on spectral reflectance than hyposalinity or shading (**Figure 4**). There was clear separation of the hypersalinity treatment seedlings along the primary ordination axis while most of the lower two salinity treatment samples were clustered. All of the Sun 50 replicates and 3 of the 4 Shade 50 replicates are separated from the other treatments on the right side of the ordination plot. Two-way crossed ANOSIM indicated that reflectance spectra for Sun and Shaded treatments were not significantly different (global *R* = -0.028, *p* = 0.569). In contrast, salinity had a significant effect on spectral reflectance (global *R* = 0.458, *p* = 0.001), with both the salinity 20 (*R* = 0.682, *p* = 0.007) and 35 (*R* = 0.464, *p =* 0.014) treatments being significantly different from the salinity 50 treatment, but not different from each other (*R* = 0.224, *p* = 0.07).

**FIGURE 5:**
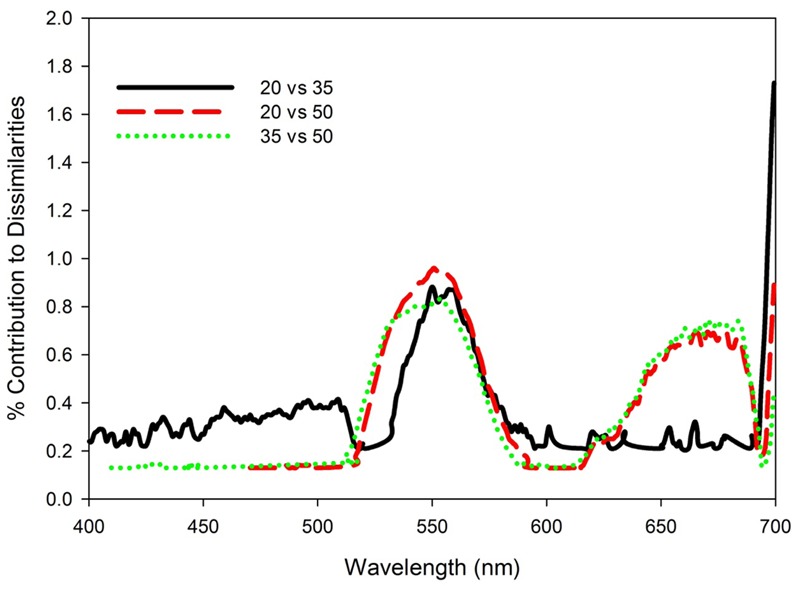
Percent contribution to dissimilarities based on pairwise SIMPER analyses between reflectance spectra for *T. testudinum* seedlings leaves after 14 days exposure to salinity 20, 35, and 50 treatments with light treatments pooled (*n* = 8).

**FIGURE 6:**
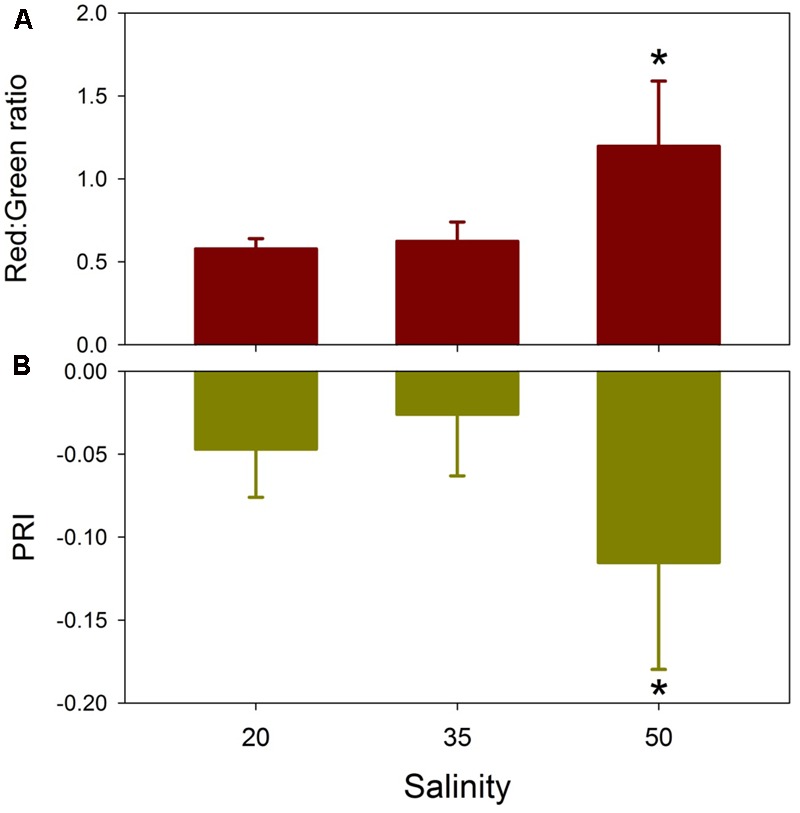
Effects of salinity on *T. testudinum* seedlings leaves after 14 days exposure to three salinity treatments (20, 35, and 50) with light treatments pooled on the Red:Green ratio **(A)** and the photochemical reflectance index (PRI) **(B)**. ^∗^indicates significant effect at *p* < 0.05 (*n* = 8).

Similarity percentage analysis of the salinity data with light treatments pooled indicated that no single wavelength contributed more than 1% to the dissimilarity among salinity treatment reflectance spectra (**Figure 5**). Consistent differences among all three salinity treatments occurred across the green wavelengths (530–580 nm), with additional discrimination between 20 versus 50 and 35 versus 50 treatments in red wavelengths (630–690 nm). The Red:Green reflectance ratio was significantly affected by salinity (*H* = 11.58, *df* = 2, *p* = 0.003), being highest in the salinity 50 treatment and not significantly different between the salinity 20 and 35 treatments (**Figure 6A**). The photochemical reflectance index (PRI) was also significantly affected by salinity (*F*_2,23_ = 8.22, *p* = 0.02) being lowest in the salinity 50 treatment and similar between the salinity 20 and 35 treatments (**Figure 6B**).

## Discussion

Leaf spectral reflectance [*R*(λ)] for *T. testudinum* seedlings exhibited a greater response to increased salinity than to low salinity or light reduction, after 2 weeks exposure. Despite a 50–70% reduction in ambient irradiance, daily mean PAR quantum flux for the Shade treatments was approximately 8 mol quanta m^-2^ day^-1^, which exceeds the daily light requirements estimated for tropical seagrasses (Chartrand et al., 2012, 2016). *R*(λ) in the Shade treatments exhibited only slight increases in the green wavelengths and small decreases in the red wavelengths. These changes are consistent with the small, but non-significant increases in chlorophyll *a* and *b* contents in the shaded seedlings relative to the full sun treatments (Howarth and Durako, 2013b). In contrast, seedlings exposed to salinity 50 conditions for 2 weeks exhibited a distinctive flattening of their *R*(λ) spectra, with reduced reflectance in the green and increased reflectance in the red. This resulted in a significant increase in the Red:Green ratios in the hypersalinity treatment relative to the control and hyposalinity treatments. Increased Red:Green ratios generally correlate with elevated anthocyanin contents (Gamon and Surfus, 1999). Leaf reddening due to increased anthocyanins is associated with numerous stressors, such as temperature extremes and high UV/visible radiation, in terrestrial plants (Chalker-Scott, 1999). Anthocyanin-induced leaf reddening has also been observed in 15 seagrass species (Novak and Short, 2010). Leaf reddening in *T. testudinum* is associated with high-light environments (Novak and Short, 2011). Although no differences in growth were observed, red-leafed *T. testudinum* shoots have shorter and narrower leaves than green-leafed shoots and higher effective quantum yields (Novak and Short, 2011). Elevated anthocyanins may function as photoprotectants or antioxidants, allowing seagrasses to maintain higher effective quantum yields after exposure to one of more stressors (Novak and Short, 2010). The significant decreases in PRI values in the salinity 50 treatment indicate reduced epoxidation of xanthophyll cycle pigments and reduced photochemical efficiency (Gamon et al., 1992). These changes in the *R*(λ) spectra and the two reflectance indices for the salinity 50 seedlings were consistent with the reduced leaf chlorophyll and carotenoid contents in these leaves (Howarth and Durako, 2013b) and the observed significant reductions in *F*_v_*/F*_m_ (Howarth and Durako, 2013a).

After 14 days at target salinities, *R*(λ) spectra, Red:Green ratios and PRI values for the salinity 20 and 35 treatment seedlings were not significantly different, reflecting similar pigments contents and photochemical efficiencies of the leaves in these two treatments (Howarth and Durako, 2013a,b). Our results are unlike a previous report of significant changes in *R*(λ) spectra for *T. testudinum* after 24 h exposure to hyposaline (16 versus 32 salinities) conditions (Thorhaug et al., 2006). These differing results may indicate that a tolerance threshold may exist for *T. testudinum* between salinities of 20 and 16. This should be tested as it could have significant management implications with respect to freshwater releases associated with CERP. Alternatively, the differing results may reflect different methods of exposure to the hyposaline conditions. Thorhaug et al. (2006) placed plants, which had been acclimated to a salinity of 32 for several days, directly into the salinity 16 treatment. This simulated a “pulsed” exposure to hyposalinity. In contrast, we gradually exposed seedlings, which were acclimated in flow-thru vaults for 4 weeks in salinities of 29–35, to changing salinities of 2 per day until our target salinities were reached. Previous studies on the seagrass *Halophila johnsonii*, which placed plants directly in treatment salinities within 24 h of collection (”pulsed” exposure) reported high mortality in both hyper- and hypo-salinity treatments (Torquemada et al., 2005; Kahn and Durako, 2008; Gavin and Durako, 2012). In contrast, gradually reducing or increasing salinity can extend seagrass salinity tolerances by approximately a salinity of 10 (Kahn and Durako, 2006; Koch et al., 2007; Griffin and Durako, 2012). Thus, the rate of change seems to be an important consideration in the resilience of seagrasses to environmental stresses.

## Conclusion

Our observations indicate that leaf spectral reflectance is a sensitive indicator of plant stress in *T. testudinum* seedlings. The results of multivariate analyses of reflectance spectra from 400 to 700 nm and statistical comparisons of two reflectance indices all suggested greater sensitivity of seedlings to hypersalinity than to hyposalinity. These results are highly consistent with previous measurements of chlorophyll fluorescence (Howarth and Durako, 2013a) and pigment content in these seedlings (Howarth and Durako, 2013b). The occurrence of extreme hypersaline conditions during the warmer seasons is thought to have played a major role in the two massive die-offs of *T. testudinum* in Florida Bay (Hall et al., 2016). Our data suggest that seedlings of this species are also highly susceptible to hypersalinity stress. Because spectral reflectance measurements are rapid, non-invasive, and have great potential use in remote sensing, they should be more widely applied for assessing stress responses of seagrasses to changing environmental conditions.

## Author Contributions

MD and JH together designed and executed the research project. MD and JH conducted the spectral reflectance measurements. MD lead the reflectance data analysis and drafted the manuscript with the assistance of JH. All co-authors commented on and approved the final manuscript draft.

## Conflict of Interest Statement

The authors declare that the research was conducted in the absence of any commercial or financial relationships that could be construed as a potential conflict of interest.
